# First evidence for temporary and permanent adhesive systems in the stalked barnacle cyprid, *Octolasmis angulata*

**DOI:** 10.1038/srep44980

**Published:** 2017-03-22

**Authors:** Fook Choy Yap, Wey-Lim Wong, Aaron G. Maule, Gerard P. Brennan, Ving Ching Chong, Lee Hong Susan Lim

**Affiliations:** 1 Institute of Biological Sciences, Faculty of Science, University of Malaya, 50603, Kuala Lumpur, Malaysia; 2 Department of Biological Science, Faculty of Science, Universiti Tunku Abdul Rahman, Jalan Universiti, Bandar Barat, 31900, Kampar, Perak, Malaysia; 3 Microbes & Pathogen Biology, The Institute for Global Food Security, School of Biological Sciences, Queen’s University Belfast, Belfast, BT9 7BL, UK

## Abstract

Although there have been extensive studies on the larval adhesion of acorn barnacles over the past few decades, little is known about stalked barnacles. For the first time, we describe the larval adhesive systems in the stalked barnacle, *Octolasmis angulata* and the findings differ from previous reports of the temporary (antennulary) and cement glands in thoracican barnacles. We have found that the temporary adhesives of cyprid are produced by the clustered temporary adhesive glands located within the mantle, instead of the specialised hypodermal glands in the second antennular segment as reported in the acorn barnacles. The temporary adhesive secretory vesicles (TASV) are released from the gland cells into the antennule via the neck extensions of the glands, and surrounded with microtubules in the attachment disc. Cement glands undergo a morphological transition as the cyprid grows. Synthesis of the permanent adhesives only occurs during the early cyprid stage, and is terminated once the cement glands reach maximum size. Evidence of the epithelial invaginations on the cement glands supports the involvement of exocytosis in the secretion of the permanent adhesives. This study provides new insight into the larval adhesives system of thoracican barnacles.

As one of the most dominant marine fouling organisms, barnacles are well-known for their efficient biological adhesive system. Barnacles have three types of adhesive systems throughout their life cycle with two occurring in the cyprid stage^1^,^2^,^3^,^4^ and one in the adult stage^5^,^6^,^7^,^8^,^9^,^10^. The two adhesive systems of cyprids, responsible for temporary and permanent adhesion^2^,^3^,^4^,^11^,^12^ play a critical role in ensuring the survival of the barnacles. This is mainly because these two types of adhesion are crucial for cyprid settlement on a suitable substratum for the next metamorphosis process^13^,^14^. Prior to the settlement efforts, the cyprid employs a pair of highly modified antennules to initiate substratum exploration, followed by close inspection before securing itself onto a suitable settlement site^15^,^16^. The temporary adhesive system of the cyprid is a reversible adhesion mechanism that allows the cyprid to rapidly attach and detach from the substratum during the exploratory phase^2^,^14^,^17^. The permanent adhesion of the cyprid involves the secretion of the “cyprid cement”, which allows the cyprid to securely attach to a suitable site on the substratum prior to metamorphosis to the adult form^1^,^18^,^19^. Since the adhesive systems of the cyprid offers spectacular underwater adhesion performance, it has the potential to inspire the development of synthetic adhesive for medical and industrial use^20^,^21^,^22^. Additionally, knowledge on the mechanism of adhesive systems in cyprids could help in solving marine biofouling issues^23^. Over the past few decades, the temporary and permanent adhesion in cyprids have been studied extensively, yet there is still a paucity of information on the synthesis and exudation processes of these two adhesives in the cyprids. To date, studies on the cyprid temporary and permanent adhesion in thoracican barnacles have mainly focused on acorn barnacles such as *Semibalanus balanoides*^1^,^2^,^12^,^15^,^24^, *Balanus amphitrite*^11^,^21^,^25^, *B. improvisus*^19^ and *Megabalanus rosa*^18^ with no published studies on the stalked barnacles.

Numerous studies on the temporary adhesion of thoracican cyprids have been carried out to understand the mechanism of reversible adhesion and most of these studies concluded that the underlying mechanism is based on chemical adhesion^2^,^12^,^14^,^23^. An earlier study suggested that cyprid temporary adhesion was a physical (suction) adhesion due to the unique appearance of the modified attachment disc^17^. However, this suction hypothesis was challenged by Nott and Foster^24^ based on their groundwork on the structure of the cyprid antennule which showed that there are only two muscles (longitudinal and transverse) present in the attachment disc. Moreover, Nott and Foster^24^ also found the presence of numerous unicellular glands in the second and third (attachment disc) segment of the antennule, which led to an alternative hypothesis that highlighted the involvement of adhesive secretion in the temporary adhesion mechanisms. This alternative hypothesis was supported by Walker and Yule^2^ who were the first to discover the deposition of the footprint material that was secreted by the cyprid for reversible temporary adhesion during the exploratory phase. The footprints of the temporary adhesive material were shown to be proteinaceous based on Coomassie brilliant blue protein dye staining^2^. Since then, the temporary adhesion has been associated with the secretion of the proteinaceous adhesives originating from the specialised hypodermal (or antennulary) glands in the second segment of the cyprid antennule.

The cyprid permanent adhesion of thoracican barnacles has stimulated much interest in the context of marine biofouling, as the permanent adhesive of the cyprids is secreted first prior to the introduction of the permanent adhesive of the adult barnacles. However, studies of the permanent adhesive system in cyprids have been progressing slowly and most references in recent studies are based on the findings of Walker^1^ who found two types of secretory cells (α and β) in the cyprid cement glands. The main components of the secretory granules in these two secretory cells have been histochemically proven to be proteinaceous, but the granules in the α-secretory cells are also found to contain phenol and enzyme polyphenol oxidase^1^. The presence of proteins, phenol and polyphenol oxidase in the cyprid permanent adhesive secretory granules has led to the hypothesis that quinnone tanning is involved in the curing of the mixed adhesive components to form the hard adhesive plaque^1^,^26^. However, the presence of phenolic amino acid was not detected in the study conducted by Hillman and Nace^27^ but instead, a small quantity of lipid was detected within the permanent adhesive. A recent study of the cyprid permanent adhesive of *B. amphitrite* supported the presence of lipid in the permanent adhesive^25^. Additionally, the cyprid permanent adhesion has proven to be a bi-phasic system containing lipid and proteins with the suggestion of lipid creating a conducive environment by removing water from the surface of the substratum before the introduction of the proteinaceous secretion^25^.

Studies of the stalked barnacle adhesion have focused solely on the adult barnacles of *Lepas anatifera*^8^ and *Dosima fascicularis*^9^,^10^. The settlement sites for these two species of stalked barnacles are very different from the acorn barnacles. Members of *Lepas* and *Dosima* are known for their pleustonic lifestyle and are usually found attached to floating objects such as driftwood, glass and plastic bottles, seaweed^28^,^29^ and even on the foam-like cement created from their own cement secretions^7^,^9^. Some of the stalked barnacle species are also found to settle on living organisms such as on the fur and feathers of penguins and seals^30^,^31^,^32^,^33^. Another group of stalked barnacles from the genus *Octolasmis* display a very unique choice of substratum for settlement as they are usually found on the branchial chamber, mouthparts and carapace of decapods^34^,^35^,^36^, on the body of seasnakes^37^, and the stem and branches of corals^38^. This indicates that members of *Octolasmis* are host-specific and need to complete their entire life-cycle within the host^39^. Therefore, it is difficult to obtain the wild cyprids of *Octolasmis* species for studies of adhesion. However, the introduction of larval *Octolasmis* species cultured under laboratory conditions has solved this issue^40^,^41^,^42^.

Here we provide the first detailed study on the adhesive systems in the cyprid of stalked barnacles, with a focus on *Octolasmis angulata*. In the present study, we investigated the development of the temporary and permanent adhesives from their respective gland cells and elucidated the exudation process for these two adhesives.

## Results

**Figure 1:**
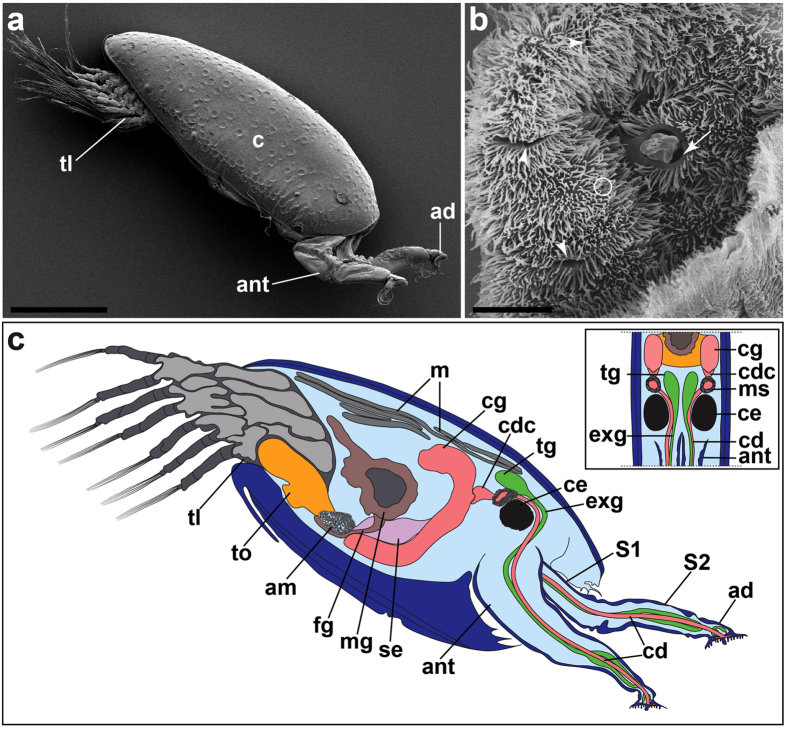
Cyprid of *Octolasmis angulata*. (**a**) Scanning electron micrograph of the whole cyprid. (**b**) Attachment disc of antennule with slit (arrowhead), small (circle) and medial (arrow) opening pores. (**c**) A schematic illustration of the cyprid temporary (green) and permanent (pink) adhesive systems. Inset: Longitudinal view of the temporary and permanent adhesive system. Note the actual number of the neck extensions of the glands in the first and second segment of the antennule is not drawn. ad, attachment disc; ant: antennule; c, cyprid carapace; cd, cement duct; cdc, collecting duct cell; ce, cyprid compound eye; cg, cement gland; exg, extension of the temporary adhesive gland; fg, foregut; m, muscle; mg, midgut; ms, muscular sac; se, supraesophageal ganglion; tg, temporary adhesive gland; tl, thoracic limbs; to, thoracic ganglion. Scale bars, 200 μm (**a**); 5 μm (**b**).

Cyprids of *Octolasmis angulata* have a pair of highly modified naupliar antennules with a distinct attachment disc at the distal segment of each antennule (Fig. 1a,b). There are three different types of openings on the surface of the attachment disc; small, slit and medial opening pores (Fig. 1b).

Two glandular systems are found associated with the cyprid adhesive systems of *O. angulata*. These two systems consist of the temporary adhesive glands and cement glands that are responsible for production of the temporary and permanent adhesives, respectively (Fig. 1c). The terminology “temporary adhesive glands” will be used in the present study as the glands are not located in the cyprid antennule.

### Temporary adhesive system: Temporary adhesive glands and associated structures

**Figure 2:**
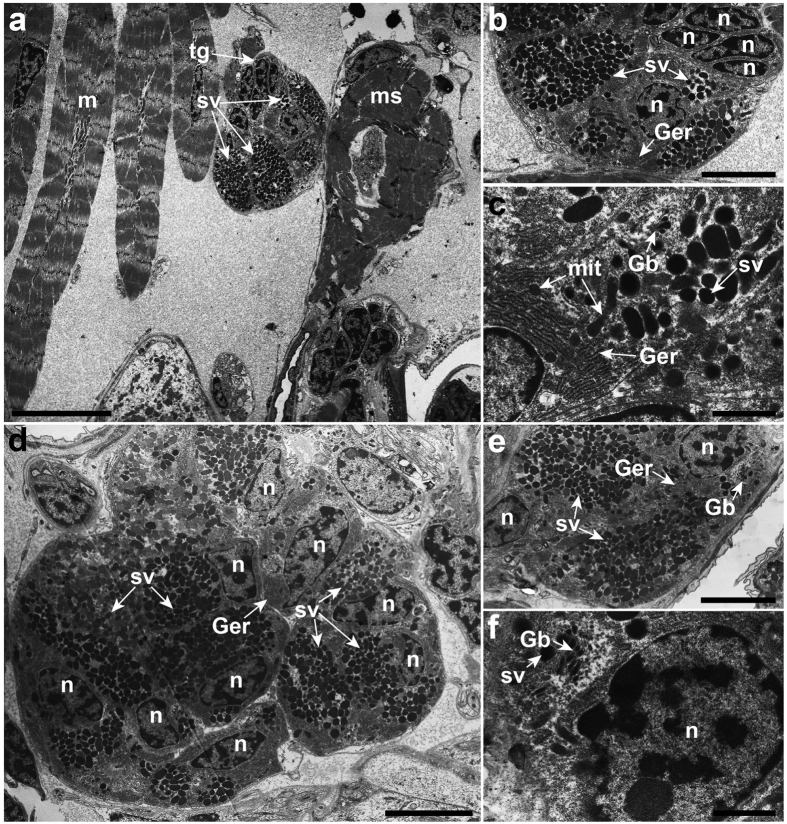
The ultrastructure of the temporary adhesive glands of one (**a**–**c**) and three-day-old cyprids (**d**–**f**) of *Octolasmis angulata*. (**a**) The oval-shaped temporary adhesive glands (tg) containing temporary adhesive secretory vesicles (sv) are located near to the dorsal muscle fibres (m) and muscular sac (ms). (**b**) Numerous temporary adhesive secretory vesicles, granular endoplasmic reticulum (Ger) and the nucleus (n) are found in the cell body of the temporary adhesive gland. (**c**) Temporary adhesive gland cell with mitochondria (mit), granular endoplasmic reticulum, Golgi bodies (Gb) and numerous temporary adhesive secretory vesicles. (**d**) The temporary adhesive gland is made up of seven to nine gland cells. (**e**) The temporary adhesive gland cell is uninucleated. (**f**) Small secretory vesicles budded off from Golgi bodies. Scale bars, 10 μm (**a**); 4 μm (**b**,**e**); 1 μm (**c**,**f**); 5 μm (**d**).

**Figure 3:**
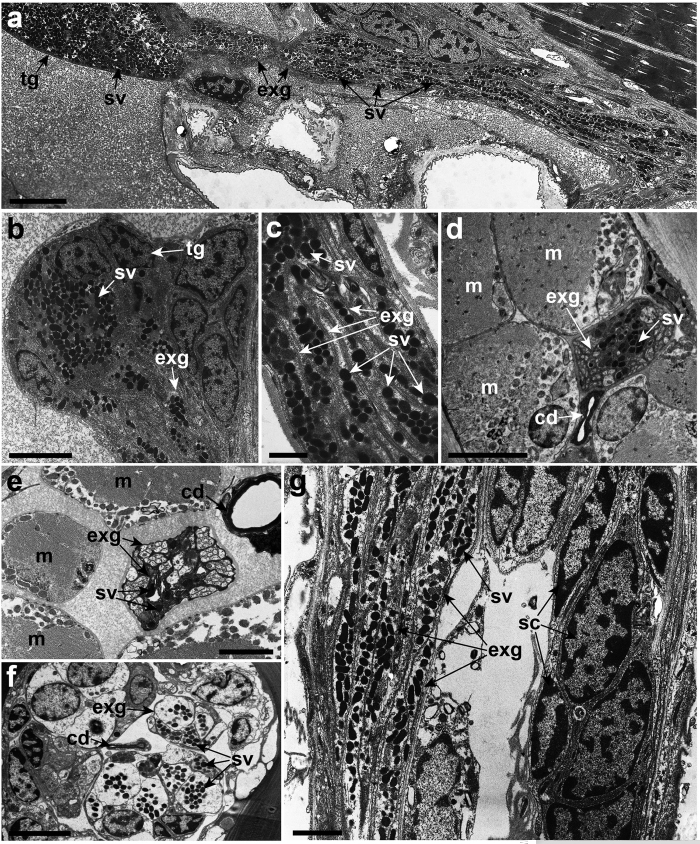
Ultrastructure of the long-necked extensions of the temporary adhesive glands of *Octolasmis angulata*. (**a**) Temporary adhesive secretory vesicles (sv) from the temporary adhesive gland (tg) entering the antennule via the extensions of the glands (exg). (**b**) Each of the gland cells has its own neck extensions of the glands to enable transport of the temporary adhesive secretory vesicles. (**c**) Extensions of the glands containing temporary adhesive secretory vesicles. (**d**) Muscle fibres (m) surrounding the extensions of the glands containing the secretory vesicles in the first segment of the antennule. Note the cement duct (cd) is surrounded by an epicuticle layer. (**e**) Cross section of the proximal region of the second segment of the antennule. The arrangement of the surrounded muscle fibres is less compact. (**f**) The distal region of the second segment of antennule showing two clusters of neck extensions of the glands and the lack of muscle fibres. (**g**) Extension of the glands in the second segment containing secretory vesicles are surrounded by numerous supporting cells (sc). Scale bars, 5 μm (**a**,**d**,**g**); 4 μm (**b**); 1 μm (**c**); 2 μm (**e**,**f**).

The temporary adhesive glands in the cyprids of *O. angulata* are located dorsal to the compound eyes (Fig. 1c). Ultrastructural examinations revealed that the temporary adhesive glands appear as oval-shaped in the one, three and five-day-old cyprids (Fig. 2a,b,d). The temporary adhesive gland consists of about seven to nine adhesive gland cells (Fig. 2a,d) with each cell body possessing dense cytoplasmic matrix, mitochondria, a well-organised granular endoplasmic reticulum, Golgi bodies and an irregularly shaped nucleus (Fig. 2a–f). The cytoplasm of the temporary adhesive gland cells contains numerous oval to elongated rod-shaped secretory vesicles ranging from 0.22–0.50 × 0.37–0.85 μm (Fig. 2a–f and Supplementary Fig. 2a,b). Small membrane-bound secretory vesicles are found at the terminal cisternae of the trans-Golgi network (Fig. 2c,e,f and Supplementary Fig. 2b). Each gland cell has its own long-necked extension (Fig. 3a) at the distal end which extends into the antennule.

Ultrastructural analysis of the longitudinal sections revealed that the adhesive secretory vesicles in the temporary adhesive glands are transported into the antennule via the long-necked extensions of the glands (Fig. 3a–c). The first segment of the antennule has 14 to 16 extensions that are clustered together and arranged medially in the antennule (Fig. 3d). This cluster of extensions is surrounded by muscle fibres and becomes more constricted (Fig. 3d). Empty extensions are occasionally observed within the first segment of the antennule (Fig. 3d).

Similar to the first segment of the antennule, the neck extensions of the temporary adhesive glands in the proximal regions of the second segment are clustered together medially and are surrounded by muscle fibres (Fig. 3e). The extensions of the glands are segregated into two or three clusters as extending distally in the second segment of the antennule (Fig. 3f). The total numbers of gland extensions in the second segment of the antennule remains similar (14 to 16) to that of the first segment. The clusters of gland extensions are surrounded by numerous supporting cells (Fig. 3g). At the distal region of the second segment of the antennule, the surrounding muscle fibres are less compactly arranged and the lumen of the gland extensions widens (Fig. 3f). Empty extensions of the glands are also found within this segment of the antennule.

**Figure 4:**
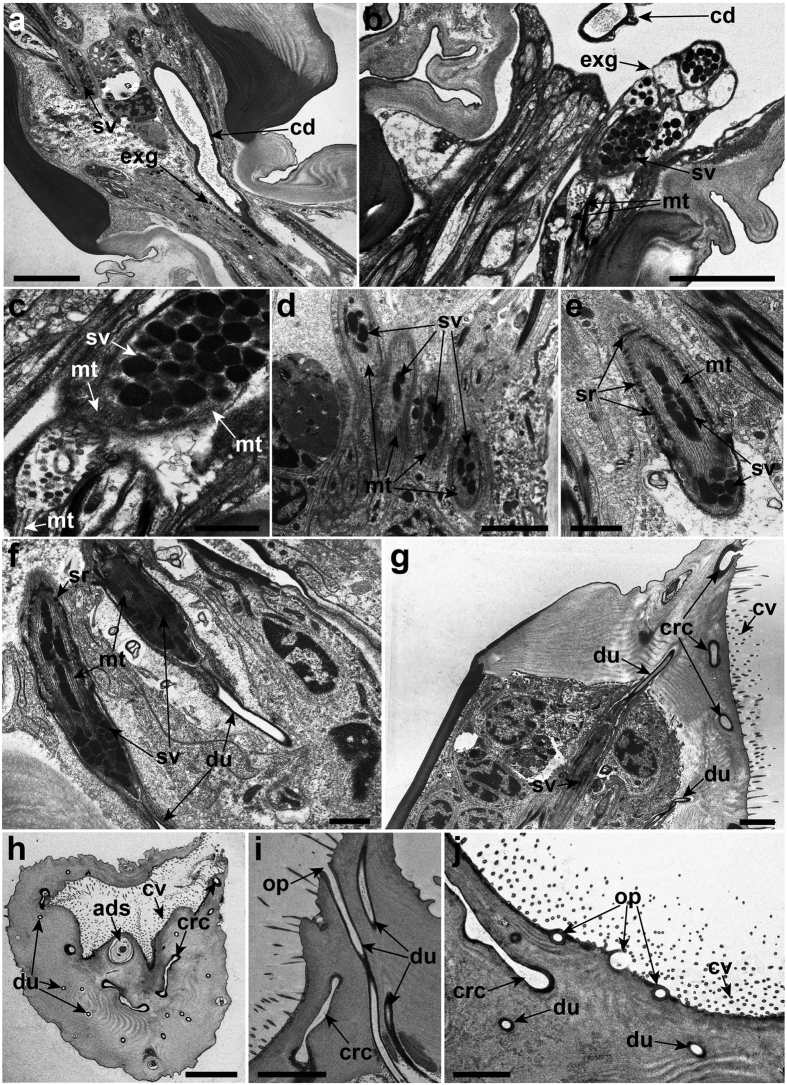
Transmission electron micrographs of the cyprid attachment disc of *Octolamis angulata*. (**a**) Temporary adhesive secretory vesicles (sv) are transported distally to the attachment disc via the extensions of the glands (exg). (**b**) Extensions of the glands containing temporary adhesive secretory vesicles passing through the narrow junctions between the second segment and the attachment disc of the antennule. (**c**) Presence of microtubule (mt) within the extensions of the glands in the attachment disc. (**d**) Secretory vesicles surrounded by microtubules within the gland extensions. (**e**) Small supporting rod-like structures (sr) surrounding the extension of the gland containing secretory vesicles. (**f**) Small ductules (du) connected at the distal end of the extension of the gland. (**g**) The ductule at the distal end penetrates the thick cuticle of the attachment disc. (**h**) A cross section of the attachment disc surface showing the random distribution of the opening pores (op) for the temporary adhesives. (**i**) Penetrated ductule connected to the opening pore. (**j**) Numerous opening pores beneath the cuticle of the attachment disc. ads, axial disc seta; cd, cement duct; crc, cement radial canal; cv, cuticular villi. Scale bars, 5 μm (**a**,**b**,**h**); 1 μm (**c**,**e**,**f**); 2 μm (**d**,**g**,**j**); 3 μm (**i**).

The neck extensions of the glands containing secretory vesicles from the second segment of the antennule extend into a narrow junction before entering the attachment disc (Fig. 4a,b). The microtubules are oriented along the longitudinal axis of the gland extensions at the narrow junction (Fig. 4c). In the attachment disc, the secretory vesicles are surrounded by the microtubules within the extensions of the glands (Fig. 4c–f). Extending toward the distal end of the attachment disc, the gland extensions are reinforced with small supporting rod-like structures at the exterior which connects to a ductule at the distal end (Fig. 4e–g). Cross-sections of the third segment of the antennule revealed that the ductules are randomly arranged on the thick cuticle surface of the attachment disc (Fig. 4h). Each ductule penetrates the thick cuticle of the attachment disc and connects to its own opening pore (Fig. 4i,j).

### Permanent adhesive system: Cement glands and associated structures

**Figure 5:**
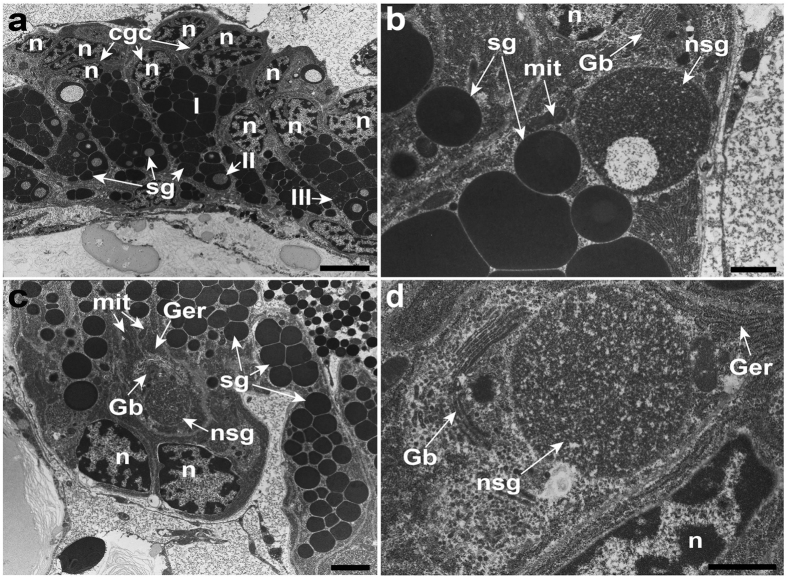
Ultrastructure of the cyprid cement glands in *Octolasmis angulata*. (**a**) Cement glands of one-day-old cyprid. Cement gland cells (cgc) contain a nucleus (n) and numerous secretory granules (sg). Note the three different types of permanent adhesive secretory granules. I, type-1; II, type-2; III, type-3. (**b**) Formation of secretory granules in the cement gland cell of one-day-old cyprid. Newly synthesised secretory granule (nsg), Golgi bodies (Gb), nucleus and mitochondria (mit) are observed within the gland cell. (**c**) Cement gland of three-day-old cyprid. Cement gland cells with a nucleus, granular endoplasmic reticulum (Ger), Golgi bodies, mitochondria and numerous secretory granules. (**d**) Condensation of the newly synthesised adhesive secretory granules in the cement glands of three-day-old cyprid. Scale bars, 5 μm (**a**); 1 μm (**b**,**d**); 2 μm (**c**).

The cyprid cement glands of *O. angulata* are located posterior to the muscular sac and compound eyes (Fig. 1c and Supplementary Fig. 1a–c). At the ultrastructural level, the cement glands consist of numerous uninucleated gland cells with an irregularly shaped nucleus lying at the proximal region of each cell body (Fig. 5a,b). A remarkably extensive granular endoplasmic reticulum (GER) occupies the cytoplasm of the gland cell (Fig. 5c,d). The remaining cytoplasm of the gland cell is filled with three different types of membrane-bound secretory granules (Fig. 5a,b). The type-1 secretory granule is most abundant and contains electron-dense matrix without any vesicles (Fig. 5a–c). The type-2 granule contains a large empty cavity (Fig. 5a,b), and the type-3 granule has more than two empty cavities within the granule (Fig. 5a). All the secretory granules are approximately 1.13–2.90 × 1.10–2.79 μm in size. In one and three-day-old cyprids, the cytoplasm of each gland cell is filled with Golgi bodies near the well-developed extensive GER (Fig. 5b–d). Small membrane-bound secretory granules are found in the vicinity of the trans-Golgi network (Fig. 5b–d). These small membrane-bound secretory granules possess a less electron-dense matrix (Fig. 5b–d). The mitochondria are oval to elongate tubiform–shaped and scattered between the organelles (Fig. 5b,c). In five-day-old cyprids, organelles such as the Golgi bodies and small membrane-bound secretory granules are not observed in the gland cells (Supplementary Fig. 2c).

**Figure 6:**
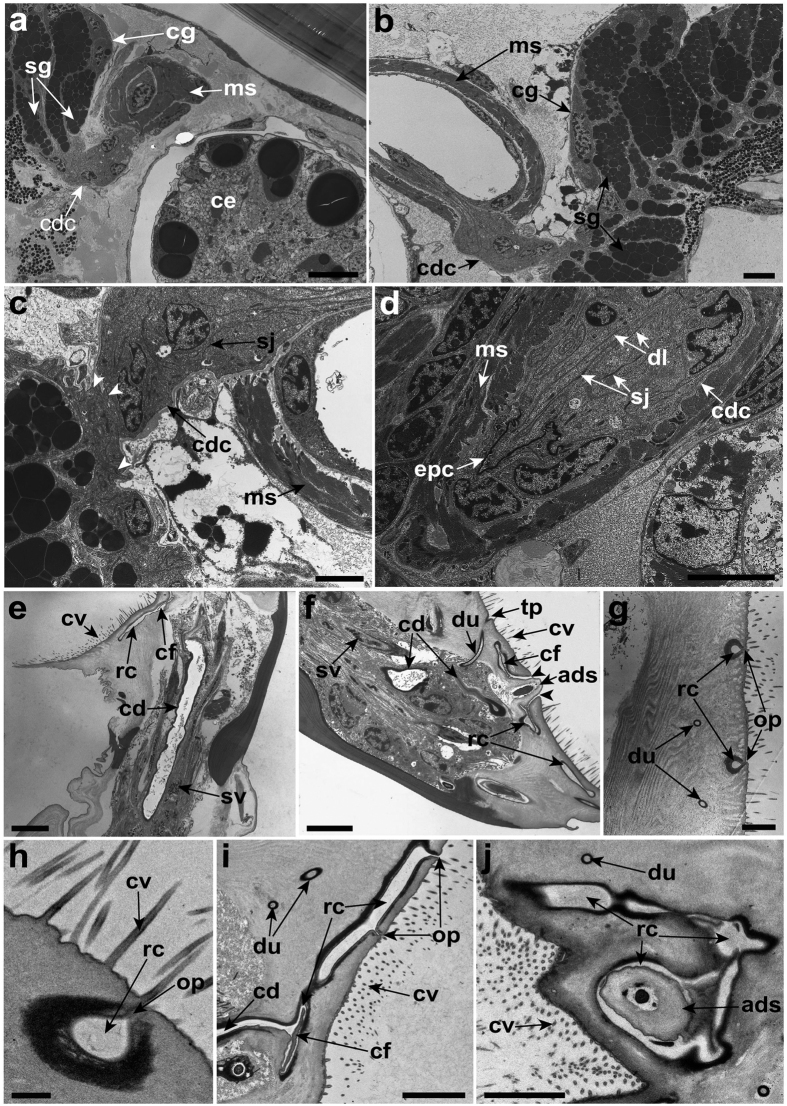
Transmission electron micrographs of the associated structures involved in the exudation of the permanent adhesive of the cyprid of *Octolasmis angulata*. (**a**) Muscular sac (ms) located anterior to the cement glands (cg) and posterior to the cyprid compound eye (ce). (**b**) Cyprid cement glands connected to the collecting duct cell (cdc) which extends into the muscular sac. (**c**) Epithelial invaginations (white arrowheads) at the distal end of the cement glands. Thin layer processes in the collecting duct cell are connected by septate junctions (sj). (**d**) Thin layer processes form the duct lining (dl) in the collecting duct cell. Note the epicuticle layer (epc) at the distal end of collecting duct cell. (**e**) Longitudinal section of the antennule. Cement duct (cd) appears as a hollow tube in the medial part of the antennule. Note the radial canal (rc) at the distal end of attachment disc and cuticular villi (cv) at the surface of the attachment disc. (**f**) Longitudinal section of the attachment disc. Radial canal connected to the medial opening pore (black arrowheads). An axial disc seta (ads) protrudes through the medial opening pore. Note the thin cuticle flaps (cf) at the proximal of the axial disc seta within the radial canal. (**g**) Radial canal connected to the opening pores (op) for the cyprid permanent adhesive secretion. (**h**) Enlarged view of the radial canal network connecting to the opening pore. (**i**) Cement duct extended into the hollow radial canal with the opening pores. (**j**) A cross-section of the attachment disc. Note the network of the radial canal. du, ductule; sv, temporary adhesive secretory vesicle; tp, temporary adhesive opening pore. Scale bars, 10 μm (**a**); 5 μm (**b**,**d**,**e**,**f**); 3 μm (**c**,**i**,**j**); 2 μm (**g**); 500 nm (**h**).

The distal end of the cement gland is connected to the collecting duct cell (Fig. 6a–c). The epithelial invagination is observed at the distal end of the cement glands before connecting to the collecting duct cell (Fig. 6c and Supplementary Fig. 3a,b). The distal end of the collecting duct cell extends into the muscular sac (Fig. 6b,c). The collecting duct cell is multinucleated and contains scattered mitochondria (Fig. 6c). This collecting duct cell possesses several thin layers of processes that form a more-or-less duct-like lining when extending into the muscular sac (Fig. 6b–d). These thin layers of processes are linked together by septate junctions (Fig. 6c,d). A dense layer of epicuticle is found lining the distal end of the collecting duct cell in the muscular sac (Fig. 6d).

The cement duct wall from the first segment of the antennule to the attachment disc is lined by a dense layer of epicuticle (Figs 3d,e,f, 4a,b and 6e,f). The cement duct lacks any organelles and it appears as a hollow tube (Figs 4a and 6e). From the ultrastructural analysis, the cement duct eventually terminates into an extensive network of the radial canal beneath the cuticle surface of the attachment disc (Fig. 6e,f,i,j). This radial canal network is linked to numerous opening pores located at the lateral and medial regions of the attachment surface (Fig. 6e–h). All these opening pores are surrounded by cuticular villi (Fig. 6g–i). Thin cuticle flaps are observed at the proximal region of the axial disc seta (Fig. 6f).

## Discussion

This study provides evidence that the cyprid temporary adhesive of *Octolasmis angulata* originates from the temporary adhesive glands located within the mantle. The cyprid temporary adhesive glands of *O. angulata* are composed of clustered unicellular glands which possess long-necked extensions leading to the small opening pores on the terminal attachment disc. The present results are at odds with previous findings by Nott and Foster^24^ who reported that the cyprid temporary adhesive is produced from the modified hypodermal glands located in the second segment of the antennule. This is probably due to the misinterpretation by Nott and Foster^24^ that the gland extension containing secretory vesicles in the second antennular segment was responsible for the production of the temporary adhesives.

It is noteworthy that the cyprid temporary adhesive glands of *O. angulata* are homologous to the small unicellular glands in the rhizocephalan barnacle cyprids, *Sacculina carcini*^43^ and *Lernaediscus porcellanae*^44^. Similar to *O. angulata*, these two species of rhizocephalan barnacles are also found to settle in the branchial chamber of their specific host crabs, but are adapted to a parasitic life-style after cyprid settlement^43^,^44^. The ancestral rhizocephalan has been suggested to be a suspension feeder^45^ as exemplified by members of thoracicans and acrothoracicans. This has led to the suggestion that *Octolasmis* may serve as an interesting model to help understand the evolution of the suspension feeders to parasitism^45^. Despite the homology of the temporary adhesive glands from the present study, it is difficult to speculate on the evolution of specialised epibiosis in *Octolasmis angulata* and the parasitism of rhizocephalan barnacles. Further detailed studies on the metamorphosis and ontogeny of *Octolasmis* are needed to help elucidate the evolution of epibiosis and parasitism in barnacles.

**Figure 7:**
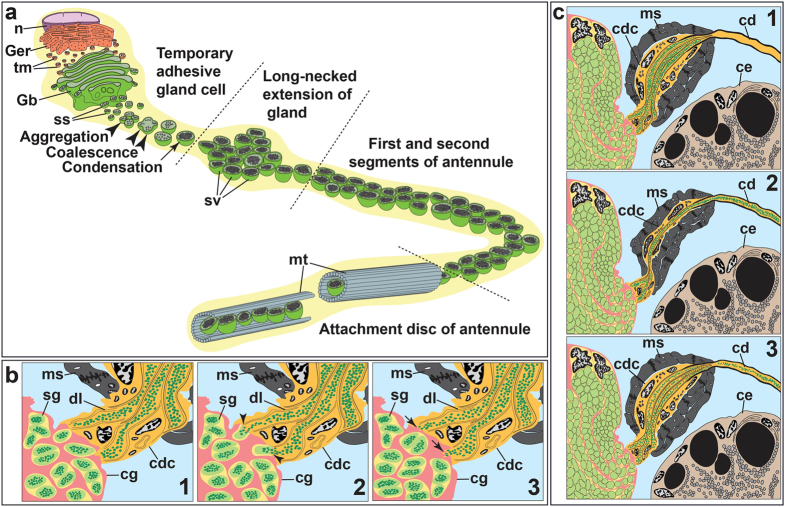
Schematic illustrations of the cyprid temporary and permanent adhesive systems in *Octolasmis angulata*. (**a**) Development of the temporary adhesive secretory vesicles (sv) in the temporary adhesive gland cell and their eventual packaging in the attachment disc. Note the synthesised small secretory vesicles from Golgi bodies undergo aggregation, coalescence and condensation to form the temporary adhesive secretory vesicles. n, nucleus; Ger, granular endoplasmic reticulum; tm, temporary adhesive material; Gb, Golgi bodies; ss, small secretory vesicles; sv, temporary adhesive secretory vesicles; mt, microtubules. (**b**) Exocytosis of the permanent adhesive secretory granules (light green, sg) from the cement gland (pink, cg) into the collecting duct (orange, cdc). (b1) Secretory granules approaching the distal end of the cement gland. (b2) Fusion of the membranes between the secretory granules and cement gland (arrowhead) leading to the release of the secretory granule contents (dark green) into the collecting duct. The distal end of the collecting duct is extended into the muscular sac (ms). The exocytosed permanent adhesive contents are transported to the distal end of the collecting duct via the duct lining (dl). (b3) Epithelial invaginations (arrow) are formed at the distal end of the cement glands. (**c**) Release of the permanent adhesives from the collecting duct into the cement duct. (c1) The exocytosed permanent adhesive contents (dark green) are delivered into the collecting duct (orange, cdc). (c2) Contraction of muscles on the muscular sac (ms) might cause compression toward the distal end of the collecting duct leading to the secretion of the permanent adhesives into the cement duct (cd). (c3) Muscular sac returns to the relaxation state and the next batch of exocytosed permanent adhesives enter the collecting duct.

Based on our ultrastructural analysis, it is apparent that the synthesis of temporary adhesives occurs within the gland cells of one, three and five-day-old cyprids in *O. angulata* (Fig. 2b,c,e,f, and Supplementary Fig. 2a,b). This indicates that the synthesis of the temporary adhesive secretory vesicles (TASV) is a continuously active process and hence, we suggest that this process might only be terminated when the cyprid permanently attaches to its substratum. The formation of TASV in the temporary adhesive gland cells of *O. angulata* begins with the synthesis of adhesive materials from the well-organised GER which is then transported into the Golgi bodies to be modified and packaged into vesicles (Fig. 7a). The vesicles will then bud off from the Golgi bodies before they aggregate, coalesce and condense to form TASV (Fig. 7a). The fully formed TASV are similar in morphology, indicating that the secretory vesicles produced by the temporary adhesive glands are homogenous (Fig. 2a–f and Supplementary Fig. 2a,b). The homogeneity of TASV has also been observed in the cyprids of the acorn barnacle, *Semibalanus balanoides*^24^ and the rhizocephalan barnacle, *S. carcini*^43^.

**Figure 8:**
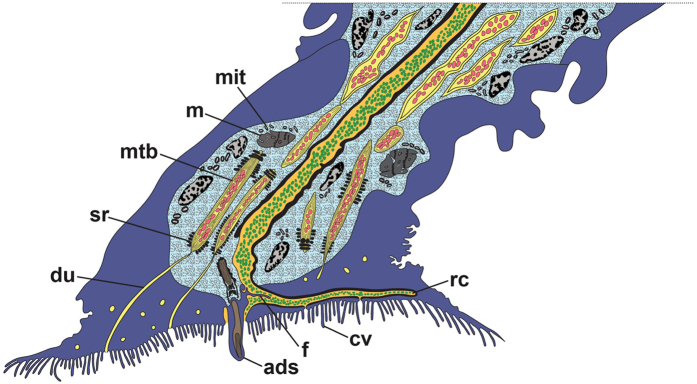
Schematic illustration of the distal end of second segment and the attachment disc based on the TEM observations of the cyprid, *Octolasmis angulata*. Temporary adhesive secretory vesicles (pink) are transported from the extensions of the glands (yellow) in the second segment of the antennule into the attachment disc. Packaging of microtubules (mtb) occurs within the extensions of the glands in the attachment disc. Extensions of the glands containing secretory vesicles with microtubules are surrounded by small supporting rod-like structures (sr) and are connected to a small ductule (du) at the distal end. The temporary adhesive secretory vesicles are exuded via the small ductule that is connected to the opening pore (d). The cyprid permanent adhesives (green) are exuded into the radial canal (rc) via the cement duct (orange). The radial canal distributes the permanent adhesive into the opening pores. A thin cuticle flap (f) is located at the proximal region of the axial disc seta (ads). m, muscle; mit, mitochondria.

The fully formed TASV will enter the extensions of the glands in the antennule. The increased number of gland extensions transporting TASV in the antennule suggests that the branching of extensions occurs when entering the first segment of the antennule. This branching of the gland extensions probably helps accelerate the transportation process of the TASV into the attachment disc. Moreover, the contraction of the surrounding muscle fibres in the first antennular segment may assist in the transportation of the secretory vesicles into the next segment of the antennule. The presence of ringlet microtubules around the secretory vesicles within the extensions of the glands in the attachment disc is most likely to assist in strengthening and transporting the secretory vesicles when entering the ductule at the distal end. Although the actual exudation of the temporary adhesives was not observed in this study, we suggest that the secretory vesicles are exuded exteriorly through the ductule that connects to the small opening pore on the attachment disc surface (Figs 4g and 8).

Most marine invertebrates such as turbellarians^46^ and echinoderms^47^ possess duo-gland adhesive systems to facilitate their temporary adhesion onto the substratum. The rapid attach and detach mechanisms in the temporary adhesion of cyprids has been suggested to involve a duo gland mechanism^48^. However, the presence of the single type of homogenous TASV and a single type of temporary adhesive gland in *O. angulata* oppose the involvement of the duo-gland mechanism; the duo-gland adhesive system requires two different gland cells to produce two different types of secretions (adhesive and de-adhesive) for the temporary adhesion behaviour^46^,^47^.

The cyprid cement glands of *O. angulata*, which secrete adhesives for permanent settlement, have undergone gradual morphological transition as the cyprid grows. The cyprid of *O. angulata* does not possess the typical kidney-shaped cement glands of thoracican barnacles, instead the cement glands appear as rod-shaped on the first day of cyprid appearance (Supplementary Fig. 1a). These rod-shaped cement glands of *O. angulata* had become slightly elongated in the three-day-old cyprid (Supplementary Fig. 1b) and eventually reached their maximum size by forming an elongated curved rod in the five-day-old cyprid (Supplementary Fig. 1c). The enlargement of cyprid cement glands is probably due to the fact that cement glands are being filled up with the adhesive secretory granules^49^ for storage purposes. Hence, the cyprid of *O. angulata* may not be able to settle on the first day of cyprid appearance as the cement glands are still immature.

Additionally, the synthesis of permanent adhesive secretory granules (PASG) is found to only occur in the cyprid cement glands of one and three-day-old cyprids. This suggests that the synthesis of PASG will be terminated once the cement glands are fully filled with secretory granules. To the best of our knowledge, this is the first report on the formation of PASG in the cyprid cement glands of stalked barnacles, exemplified here by *O. angulata*. The synthesis of PASG in the cement gland cells is relatively similar to the synthesis of TASV in the temporary adhesive gland cells, which involves organelles such as GER and Golgi bodies in synthesising, modifying and packaging the adhesive materials into small granules. These small granules begin to aggregate, coalesce and condense during the formation of PASG. Unlike TASV in the temporary adhesive glands, the fully developed PASG from the cement glands are not transported directly into the cement duct in the antennule and instead, are transported into the columnar-shaped pouch of the gland cell for storage purposes. In *O. angulata*, the cyprid cement glands produce three different types of adhesive secretory granules that are distinct from the α and β- secretory granules found in *S. balanoides*^1^.

The actual exudation process of the cyprid permanent adhesive in *O. angulata* was not observed in the present study, but the observations strongly support the prevailing hypothesis about the involvement of exocytosis in the release of PASG from the cement glands into the collecting duct^18^,^19^. This is supported by the presence of epithelial invaginations at the distal end of the cement glands (Fig. 6c and Supplementary Fig. 3a,b). These epithelial invaginations are probably formed from the fusion of the membranes between the PASG and cement glands, which may lead to release of the permanent adhesive content into the collecting duct (Fig. 7b,c). Previously, the collecting duct was postulated to be present at the medial region of the cement glands which is connected to the muscular sac^50^. However, this arrangement of median collecting duct is not observed in *O. angulata* and instead, the collecting duct is found connected to the invaginations of the cyprid cement glands with the distal end of the collecting duct extending into the muscular sac (Fig. 6b,c). Hence, the exocytosed permanent adhesive contents are postulated to be transported into the distal end of the collecting duct via the duct lining that is formed by the thin layer processes (Fig. 7b,c). Similar to previous studies^18^,^50^, the contraction of the muscular sac may serve as a “pump” to transport the permanent adhesive contents from the collecting duct into the cement duct (Fig. 7c). The cement duct of *O. angulata* probably extends into the muscular sac with the proximal region of the duct protruding into the distal end of the collecting duct (Figs 6d and 7c). This is proposed because the epicuticle layer that lines the cement duct is found at the distal end of the collecting duct (Fig. 6d). The function of the epicuticle layer on the cement duct is probably to strengthen the structure of the duct and prevent it from collapsing during contraction of the muscular sac which may create high pressure resulting in the secretion of permanent adhesives into the cement duct. As noted earlier, the cement duct in the antennule is connected to the radial canal at the distal end of the attachment disc, suggesting that the function of the radial canal is to distribute the permanent adhesive secretions into the opening pores (Fig. 8). It is noteworthy that the presence of thin cuticle flaps at the proximal region of the axial disc seta may not only act as a valve in preventing backflow of the permanent adhesive secretions or sea water but may also help in distributing permanent adhesives into the slit opening pores before entering the medial opening pore.

In conclusion, we have documented for the first time the glands that are responsible for the cyprid temporary and permanent adhesive secretions in *O. angulata* based on the ultrastructural studies. Our study revealed that the cyprid temporary and permanent adhesive glands of *O. angulata* are different from those of the acorn barnacles, but are rather similar to the rhizocephalan barnacles. This study has clarified the synthesis process of TASV in the temporary adhesive glands and PASG in the cement glands and helps elucidate the development of these two adhesive glands. In future, chemical analyses of the cyprid temporary and permanent adhesives in *O. angulata* are needed to determine the differences in the temporary and permanent adhesives of stalked and acorn barnacles.

## Methods

### Larvae culture

Barnacle larvae of *Octolasmis angulata* were cultured under laboratory conditions^42^. About 20 adult *O. angulata* individuals were removed from the freshly excised gills of the mud crabs, *Scylla tranquebarica* obtained from Kampung Sungai Tiang, Perak, Malaysia using fine forceps and a needle. Adult barnacles were cultured in a glass bowl containing 0.45 μm filtered sea water of approximately 33 ppt at 27 °C. Adult *O. angulata* were fed with freshly hatched brine shrimp, *Artemia* sp. (East Ocean Aquatic, Singapore). The nauplii released from the adult barnacles were cultured in sterile Petri dishes containing filtered sea water. The larval culture was maintained at 27 °C in a 12:12 h light-dark cycle. The barnacle nauplii were fed with cultured microalgae, *Pavlova lutheri* and *Tetraselmis sueicica* (UTAR Microalgae Sdn Bhd, Malaysia) until the nauplii reached the cyprid stage. The exploratory behaviour of the cyprids was observed under the stereomicroscope.

### Scanning electron microscopy

The cyprids (n = 6) were relaxed in 8% magnesium chloride^51^,^52^,^53^ for 1 h, washed in 0.45 μm filtered sea water and then fixed with 2.5% glutaraldehyde (sea water base) for 1 h. The cyprids were dehydrated through an ascending series of ethanol, critical point dried, mounted on aluminium stubs and sputter-coated with gold. The coated cyprids were viewed using a Hitachi SU8000 field-emission scanning electron microscope operating from 5 to 15 kV.

### Transmission electron microscopy

One, three and five-day-old cyprids (each samples, n = 4) were relaxed with 8% magnesium chloride^51^,^52^,^53^ for 1 h and washed thoroughly with 0.45 μm filtered 33 ppt sea water. The cyprids were then fixed with modified Karnovsky (2% paraformaldehyde and 2.5% double-distilled glutaraldehyde buffered with 0.1 M sodium cacodylate containing 3% sucrose, pH 7.4) overnight at 4 °C. The specimens were washed with cacodylate buffer, post-fixed with 1% aqueous osmium tetroxide, OsO_4_, washed again with cacodylate buffer, dehydrated through a graded series of ethanol, infiltrated and embedded in Agar epoxy Resin 100 (Agar Scientific). The embedded specimens were polymerised for 48 h at 60 °C. Semithin sections were cut with glass knives on a Reichert Ultracut E ultramicrotome (Reichert-Jung) to locate the target structures. The semithin sections were stained with toluidine blue and examined under Leica DMRB microscope equipped with the Leica QWin Plus software (Leica, Wetzlar,Germany). Serial ultrathin sections (60–90 nm) were cut with diamond knives (Diatome, Switzerland). The ultrathin sections were collected on bare 75, 100 and 150-mesh copper grids, double stained with uranyl acetate (for 8 min) and lead citrate^54^ (for 5 min) and examined with FEI CM100 and Hitachi ST7700 transmission electron microscopes operating at 80 and 100 kV.

## Additional Information

**How to cite this article:** Yap, F. C. *et al*. First evidence for temporary and permanent adhesive systems in the stalked barnacle cyprid, *Octolasmis angulata. Sci. Rep.* **7**, 44980; doi: 10.1038/srep44980 (2017).

**Publisher's note:** Springer Nature remains neutral with regard to jurisdictional claims in published maps and institutional affiliations.

## Supplementary Material

Supplementary Information
