# Supplementary material for: First evidence for temporary and permanent adhesive systems in the stalked barnacle cyprid, Octolasmis angulata
Source: Sci Rep. 2017 Mar 22;7:44980. doi: 10.1038/srep44980 (PMC5361150; doi:10.1038/srep44980)
Supplement: Supplementary Information [file srep44980-s1.pdf]

First evidence for temporary and permanent adhesive systems in the stalked barnacle cyprid, *Octolasmis angulata*

Fook Choy Yap[1], Wey-Lim Wong[2*], Aaron G. Maule[3], Gerard P. Brennan[3], Ving Ching Chong[1] and Lee Hong Susan Lim[1,4]

[1] Institute of Biological Sciences, Faculty of Science, University of Malaya, 50603 Kuala Lumpur, Malaysia.

[2] Department of Biological Science, Faculty of Science, Universiti Tunku Abdul Rahman, Jalan Universiti, Bandar Barat, 31900 Kampar, Perak, Malaysia. Phone: Tel: + 605 468 8888 ext. 4664. Fax: + 605 466 1676. Email: wongwl@utar.edu.my (Corresponding author)

[3] Microbes & Pathogen Biology, The Institute for Global Food Security, School of Biological Sciences, Queen's University Belfast, Belfast BT9 7BL, UK

[4] Deceased

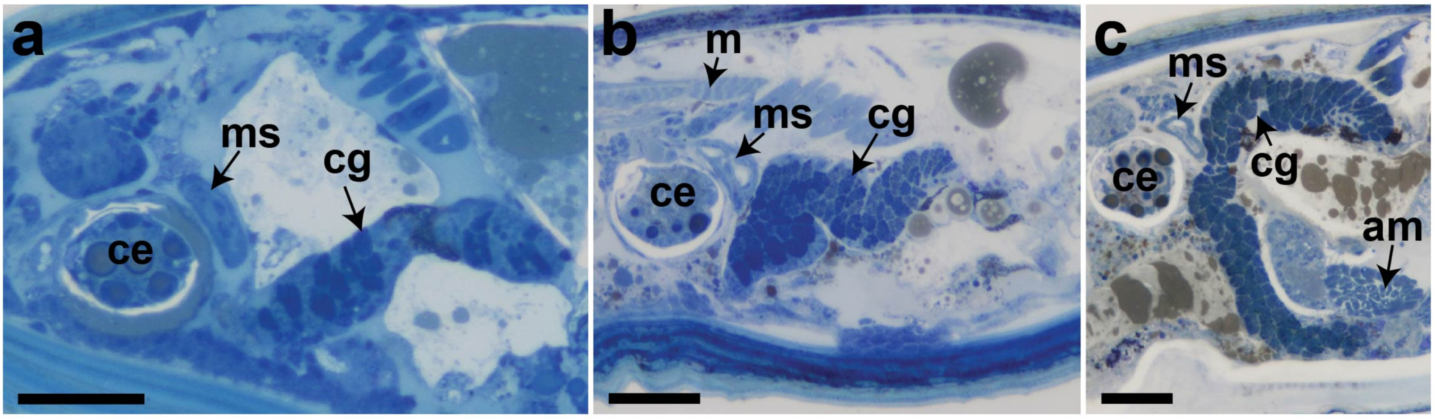

Supplementary Figure 1

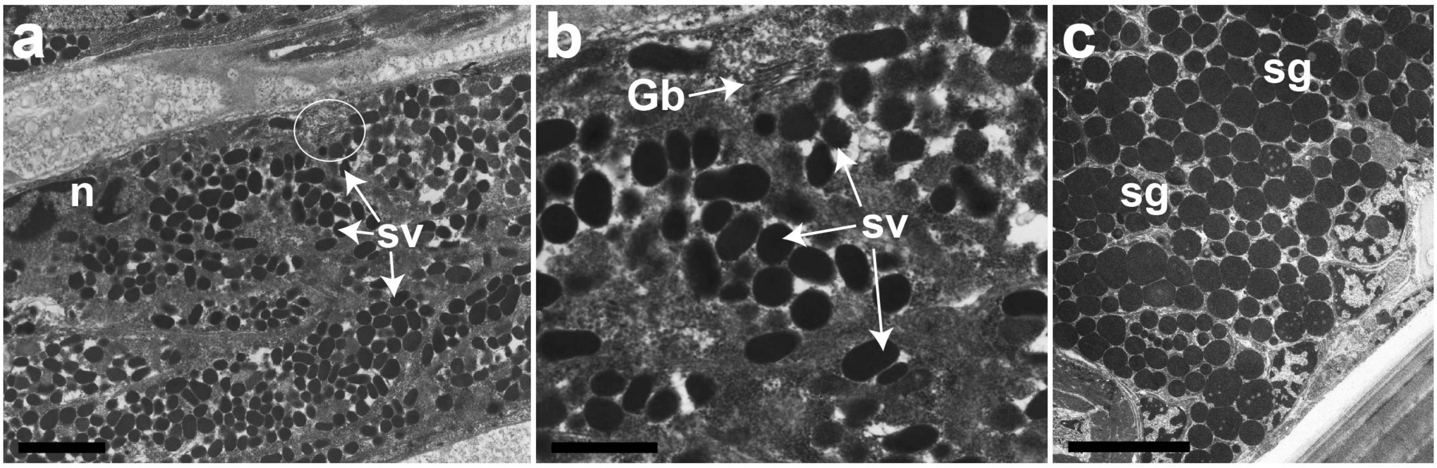

Supplementary Figure 2

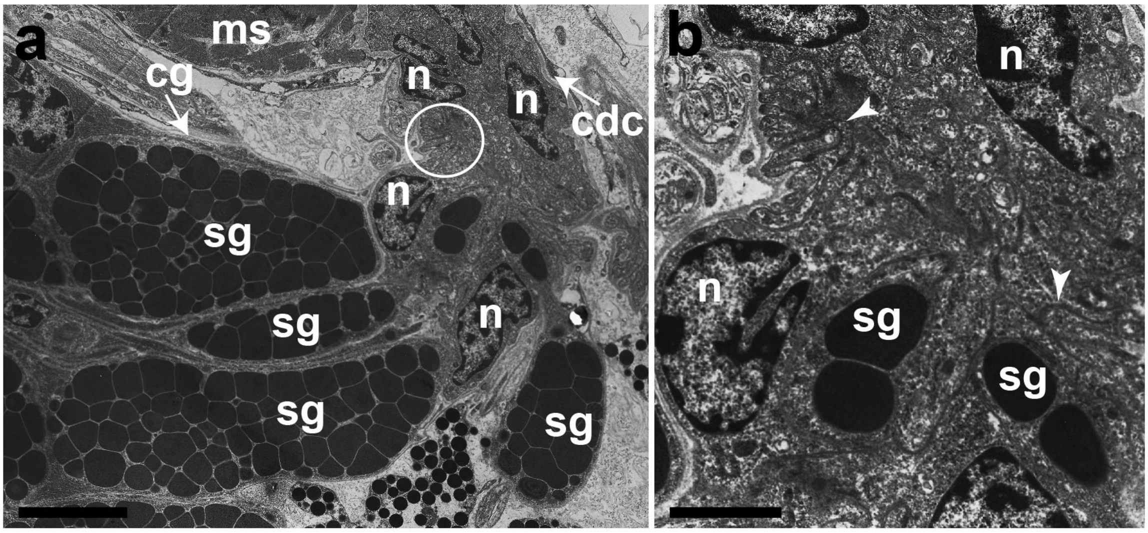

Supplementary Figure 3

Supplementary Figure Legends

Supplementary Figure 1. The morphology of the cyprid cement glands of *Octolasmis* angulata in one, three and five-day-old. (a) Cement gland (cg) appears as rod shaped in one-day-old cyprid. (b) Rod-shaped cement gland becomes slightly elongated in three-day-old. (c) Cyprid cement gland reach its maximum size and formed the elongated curved rod-shaped. ce, compound eye; ms, muscular sac; m, muscle; am, adductor muscle. Scale bars, 50 µm (a-c).

Supplementary Figure 2. Temporary adhesive glands and cement glands of the five-day-old cyprid of *Octolasmis angulata.* (a) Temporary adhesive glands with a nucleus (n) and numerous temporary adhesive secretory vesicles (sv). (b) Enlarged view of the circled area of (a). Synthesis of the temporary adhesive secretory vesicles occurs in the gland cells. Small secretory vesicles budded off from the Golgi bodies (Gb). (c) Cement gland is completely filled with developed permanent adhesive secretory granules (sg). Scale bars, 2 µm (a); 1 µm (b); 5 µm (c).

Supplementary Figure 3. Ultrastructure of the distal end of the cyprid cement gland and collecting duct of *Octolasmis angulata*. (a) Permanent adhesive secretory granules (sg) moving toward the collecting duct (cd). Note the epithelial invaginations (circled). (b) Enlarged view of the circled area of (a). The collecting duct connected to the epithelial invaginations (arrowhead) at the distal end of the cement glands. cg, cement gland; n, nucleus; ms, muscular sac. Scale bars, 5 µm (a); 2 µm (b).
